# Supplementary material for: A histomorphological atlas of resected mesothelioma discovered by self-supervised learning from 3446 whole-slide images
Source: Nat Commun. 2025 Oct 7;16:8891. doi: 10.1038/s41467-025-63846-9 (PMC12504713; doi:10.1038/s41467-025-63846-9)
Supplement: Supplementary file 1 — Supplementary Information [file 41467_2025_63846_MOESM1_ESM.pdf]

Option 1 - Epithelioid tumour							
	Topic	Options					
1	Predominant architectural growth pattern	Tubular	Papillary	Trabecular	Adenomaotoid	Solid	Micropapillary
2	Second most predominant pattern	Tubular	Papillary	Trabecular	Adenomaotoid	Solid	Micropapillary
3	Nuclear atypia	Mild	Moderate	Severe			
4	Inflammation*	None-sparse	Mild-moderate	Marked			
5	Necrosis**	None	Some	Universal			
6	Stroma:Tumour ratio	More stroma	Roughly equal	More tumour			
7	Stromal cellularity***	Low	Moderate	High			
8	ˆBiphasic features?	No	Yes				
9	Other notable features or comments	free text/annotations					
	ˆBy biphasic, we refer to mixed epithelioid and spindled morphology within individual tiles rather than within the cluster						

Option 2 - Spindle cells / extracellular matrix				
	Topic	Options		
1	Cellularity	Low	Medium	High
2	Architecture	Orderly (parallel)	Less orderly (eg wavy)	Disorderly (random/storiform)
3	Presence of desmoplastic sarcomatoid morphology	Present	Absent	
4	Nuclear atypia	None/mild	Moderate	Severe
5	Inflammation*	None-sparse	Mild-moderate	Marked
6	Necrosis**	None	Some	Universal
7	ˆBiphasic features?	No	Yes	
8	Other notable features or comments	free text/annotations		
	ˆBy biphasic, we refer to mixed epithelioid and spindled morphology within individual tiles rather than within the cluster			

Option 3 - Non-tumour								
Topic	Options							
Tiles contain mostly (by area)	Normal/near-normal lung			Collagenosis	Haemorrhage	Vessels (wall or lumen)	Airway	Cartilage
Second most common feature (by area)	Normal/near-normal lung	Non-neoplastic pathological lung	Elastosis	Collagenosis	Haemorrhage	Vessels (wall or lumen)	Airway	Cartilage
Inflammation*	None-sparse	Mild-moderate	Marked					
Necrosis**	None	Some	Universal					
Other notable features or comments	free text/annotations							
Inflammation*	None-sparse	Mild-moderate	Marked					

Supplementary Table 1: Criteria for Pathologist Annotation Sheets

Leiden Resolution	Dataset	Balanced Accuracy	AUC	Sensitivity	Specificity
2.0	LATTICe-M	0.79 ± 0.05	0.88 ± 0.04	0.81 ± 0.1	0.77 ± 0.09
4.0		0.79 ± 0.04	0.89 ± 0.03	0.81 ± 0.1	0.77 ± 0.06
7.0		0.79 ± 0.04	0.88 ± 0.04	0.78 ± 0.06	0.79 ± 0.08
CLAM		0.79 ± 0.03	0.87 ± 0.04	0.77 ± 0.09	0.80±0.05
2.0	TCGA-MESO	0.71 ± 0.03	0.8 ± 0.03	0.63 ± 0.06	0.79 ± 0.04
4.0		0.78 ± 0.05	0.85 ± 0.02	0.7 ± 0.05	0.85 ± 0.06
7.0		0.78 ± 0.05	0.85 ± 0.04	0.67 ± 0.08	0.89 ± 0.04
CLAM		0.67 ± 0.05	0.74 ± 0.01	0.55 ± 0.04	0.79 ± 0.03

Supplementary Table 2: Subtype classification performance metrics across clustering resolutions and datasets. Balanced accuracy, area under the curve (AUC), sensitivity, and specificity scores for mesothelioma subtype classification using HPL (across different Leiden clustering resolutions) and CLAM methods on LATTICe-M and TCGA-MESO cohorts. Scores represent means from 5-fold cross-validation following data preprocessing with the edited nearest neighbour (ENN) sampling method in HPL.

	HPCs			Clinical and HPCs	
Leiden Resolutions	Train	Test	TCGA	Train	Test
2.0	0.67 ± 0.0	0.65 ± 0.03	0.65 ± 0.01	0.68 ± 0.01	0.66 ± 0.03
4.0	0.69 ± 0.0	0.66 ± 0.03	0.66 ± 0.02	0.7 ± 0.0	0.66 ± 0.03
7.0	0.7 ± 0.01	0.65 ± 0.04	0.66 ± 0.01	0.71 ± 0.01	0.66 ± 0.04
	Risk Score			Clinical and Risk Score	
CLAM	0.60±0.01	0.60±0.04	0.61±0.0	0.62±0.02	0.62±0.05

Supplementary Table 3: Concordance indices for patient outcome prediction across datasets and clustering resolutions. Patient outcome prediction was performed using Cox proportional hazards models, with concordance indices reported across training, testing (LATTICe-M), and external validation (TCGA-MESO) datasets at different Leiden clustering resolutions using 5-fold cross-validation. For the primary dataset, clinical variables (tumour type, TNM stage, and age) were integrated with patient vectors derived from HPL. Additionally, survival analysis was conducted independently using the CLAM multiple instance learning approach, where risk scores were generated through gated attention mechanisms.

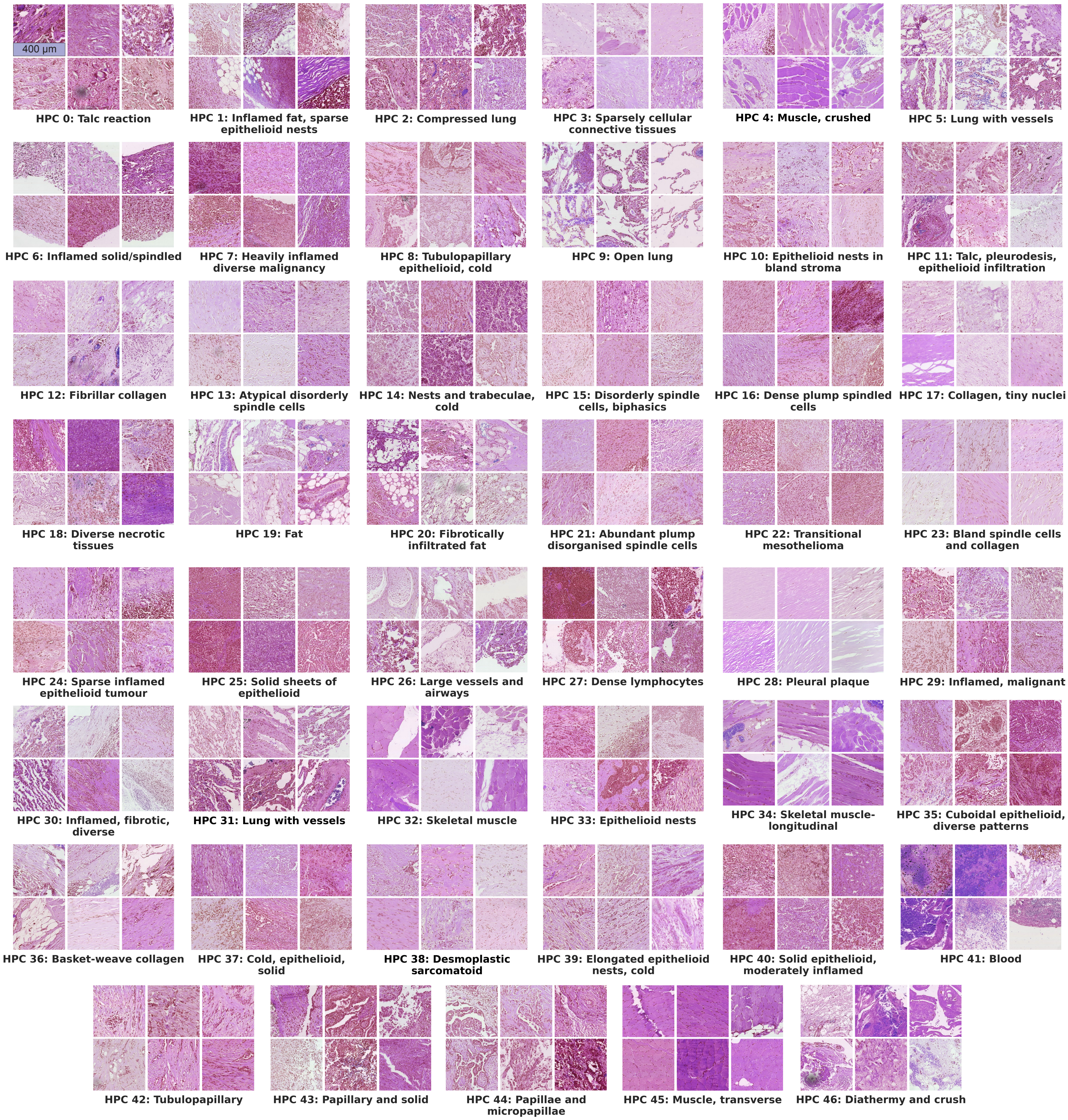

Supplementary Figure 1: Representative of all HPC tiles in the LATTICe-M dataset. Examples of tiles from LATTICe-M dataset assigned to all HPCs with short annotations provided from expert pathologists (scale bar, 400 µm).

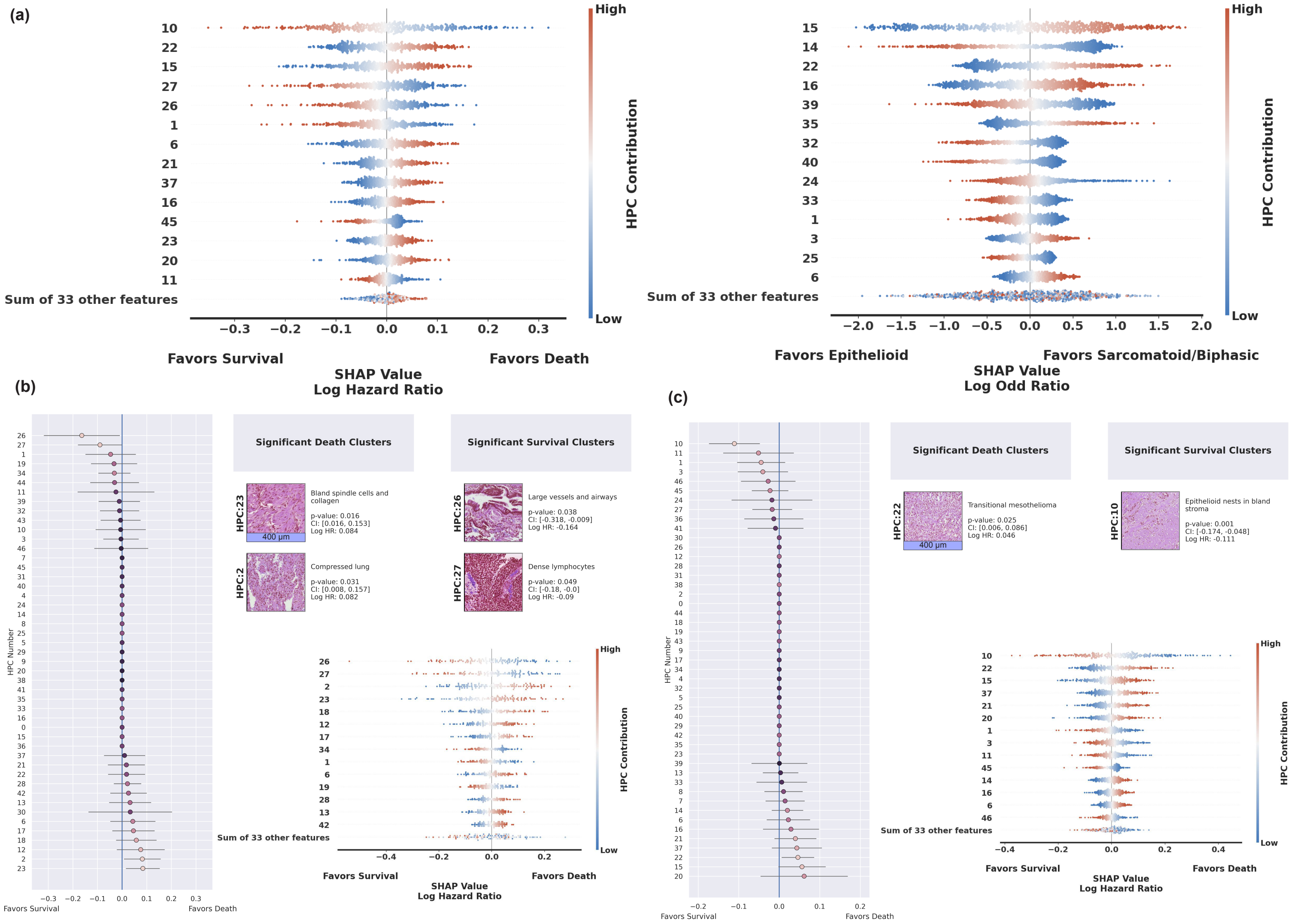

Supplementary Figure 2: SHAP analysis and subtype-specific Cox model results a SHAP plots showing the impact of individual HPCs on model predictions for overall survival (left - n= 512) and subtype classification (right - n=3446), ordered by mean absolute SHAP value. **b,c** SHAP summary plots and forest plots from Cox models trained separately on Epithelioid and Sarcomatoid/Biphasic group cases, highlighting subtype-specific prognostic HPCs. All SHAP analyses were performed on case-independent test sets. SHAP values represent each feature's impact on the model's output. Forest plots show log hazard ratios (center) with 95% confidence intervals (error bars), from subtype-specific Cox models. The plots are organized with Sarcomatoid/Biphasic on the left and Epithelioid on the right (scale bar, 400 μm).

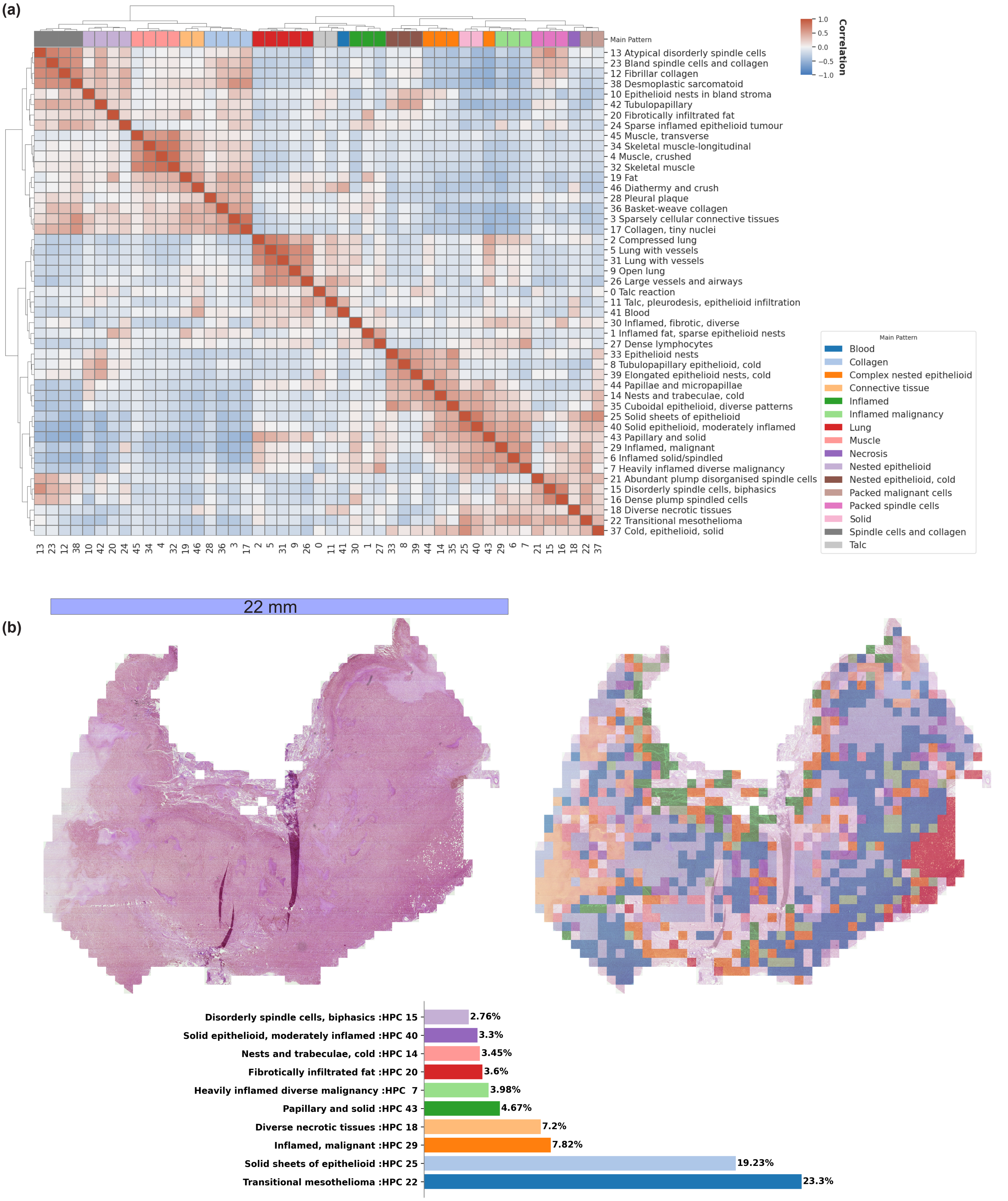

Supplementary Figure 3: HPC correlation analysis and epithelioid case example. **a** HPC Pearson (two-sided) correlation heatmap; annotations were independently provided by a pathologist after examination of representative sets of tile images (100 random tiles per HPC). **b** An epithelioid sample case characterised mostly by transitional mesothelioma HPC (scale bar, 22 mm).
