# Supplementary material for: A histomorphological atlas of resected mesothelioma discovered by self-supervised learning from 3446 whole-slide images
Source: Nat Commun. 2025 Oct 7;16:8891. doi: 10.1038/s41467-025-63846-9 (PMC12504713; doi:10.1038/s41467-025-63846-9)
Supplement: Supplementary file 3 — Reporting Summary [file 41467_2025_63846_MOESM3_ESM.pdf]

nature portfolio

Corresponding author(s): John Le Quesne
Ke Yuan

Last updated by author(s): Aug 1, 2025

Reporting Summary

Nature Portfolio wishes to improve the reproducibility of the work that we publish. This form provides structure for consistency and transparency in reporting. For further information on Nature Portfolio policies, see our Editorial Policies and the Editorial Policy Checklist.

Statistics

For all statistical analyses, confirm that the following items are present in the figure legend, table legend, main text, or Methods section.

n/a	Confirmed	
☐	☒	The exact sample size (*n*) for each experimental group/condition, given as a discrete number and unit of measurement
☐	☒	A statement on whether measurements were taken from distinct samples or whether the same sample was measured repeatedly
☐	☒	The statistical test(s) used AND whether they are one- or two-sided<br>*Only common tests should be described solely by name; describe more complex techniques in the Methods section.*
☒	☐	A description of all covariates tested
☒	☐	A description of any assumptions or corrections, such as tests of normality and adjustment for multiple comparisons
☐	☒	A full description of the statistical parameters including central tendency (e.g. means) or other basic estimates (e.g. regression coefficient) AND variation (e.g. standard deviation) or associated estimates of uncertainty (e.g. confidence intervals)
☐	☒	For null hypothesis testing, the test statistic (e.g. *F*, *t*, *r*) with confidence intervals, effect sizes, degrees of freedom and *P* value noted<br>*Give P values as exact values whenever suitable.*
☒	☐	For Bayesian analysis, information on the choice of priors and Markov chain Monte Carlo settings
☒	☐	For hierarchical and complex designs, identification of the appropriate level for tests and full reporting of outcomes
☒	☐	Estimates of effect sizes (e.g. Cohen's *d*, Pearson's *r*), indicating how they were calculated

Our web collection on statistics for biologists contains articles on many of the points above.

Software and code

Policy information about availability of computer code

Data collection	REDCap (Research Electronic Data Capture)
Data analysis	Python 3.8.17<br>TensorFlow 1.15<br>Cudatoolkits 10.0<br>Cudnn 7.6.0<br>(other libraries used are provided in requirement.txt file on https://github.com/FarzanehSeyedshahi/Histomorphological-Phenotype-Learning/blob/master/requirements.txt)

For manuscripts utilizing custom algorithms or software that are central to the research but not yet described in published literature, software must be made available to editors and reviewers. We strongly encourage code deposition in a community repository (e.g. GitHub). See the Nature Portfolio guidelines for submitting code & software for further information.

Data

Policy information about availability of data

All manuscripts must include a data availability statement. This statement should provide the following information, where applicable:

- Accession codes, unique identifiers, or web links for publicly available datasets
- A description of any restrictions on data availability
- For clinical datasets or third party data, please ensure that the statement adheres to our policy

The LATTICe cohort (histology whole slide images and clinical data) used in this study is not publicly available due to their extremely large size and ethical limitations according to the LATTICe agreement, which makes public hosting technically impractical. However, we are delighted to make the data available for academic research purposes upon request. Interested researchers may contact the corresponding author via the email provided. Access will be granted for a limited period based on a clear research purpose and mutual agreement, with data use restricted to non-commercial research. We aim to respond to access requests as soon as possible. TCGA mesothelioma RNAseq data has been retrieved from UCSC Xena [https://xenabrowser.net/datapages/?cohort=GDC%20TCGA%20Mesothelioma%20(MESO)] and images from Genomic Data Commons (GDC) portal [https://www.cancer.gov/ccg/research/genome-sequencing/tcga/studied-cancers/mesothelioma-study]. St. George Hospital TMA Dataset is available on MesoGraph GitHub [https://github.com/measty/MesoGraph].

Research involving human participants, their data, or biological material

Policy information about studies with human participants or human data. See also policy information about sex, gender (identity/presentation), and sexual orientation and race, ethnicity and racism.

Reporting on sex and gender	The LATTICe-M dataset included both genders (436 male and 76 female), reflecting the gender distribution of mesothelioma diagnoses and surgeries at Leicester, where the majority of cases were male at that time. Sex and gender were reported in the study; however, no sex-based analysis was performed with the aim of training a self-supervised model.
Reporting on race, ethnicity, or other socially relevant groupings	Race, ethnicity, or other socially relevant groupings were not reported or used in this study.
Population characteristics	The LATTICe-M dataset comprised 512 patients, with ages ranging from 36 to 85 years (median: 64.5 years) at the time of surgery. Information on smoking history, weight, and height was incomplete and not utilised in the analysis. A subset of 485 patients with sufficient sample data was selected for model training.
Recruitment	*Describe how participants were recruited. Outline any potential self-selection bias or other biases that may be present and how these are likely to impact results.*
Ethics oversight	The LATTICeM cohort includes human tissue samples collected post-surgery, along with corresponding clinical data. Initial cohort construction was approved under East Midlands Research Ethics Committee. Ongoing resource governance is managed by the Greater Glasgow and Clyde Biorepository under an amendment approved by the Leicester South REC. All samples were fully anonymized prior to analysis. Patient clinical data were used solely for research purposes under ethical oversight, with no identifiable information accessed.

Note that full information on the approval of the study protocol must also be provided in the manuscript.

Field-specific reporting

Please select the one below that is the best fit for your research. If you are not sure, read the appropriate sections before making your selection.

☒ Life sciences ☐ Behavioural & social sciences ☐ Ecological, evolutionary & environmental sciences

For a reference copy of the document with all sections, see nature.com/documents/nr-reporting-summary-flat.pdf

Life sciences study design

All studies must disclose on these points even when the disclosure is negative.

Sample size	The whole LATTICe-M dataset comprised 512 patients. No formal power calculation was performed; however this number is well above the number of cases routinely used in cancer studies. Furthermore, the data collection is by some way the largest imageset in this disease type which has been used for AI training.
Data exclusions	27 patients were excluded from the model training for having insufficient tumour image tiles (<100 tiles).
Replication	Our model was tested on an external image dataset from TCGA, confirming model performance in both tumour subtyping and outcome prediction, and on a further TMA derived image dataset, validating performance in tumour subtyping.
Randomization	No randomization was required in this study.
Blinding	Our external pathologist panel were blinded to HPC details and to patient data.

Reporting for specific materials, systems and methods

We require information from authors about some types of materials, experimental systems and methods used in many studies. Here, indicate whether each material, system or method listed is relevant to your study. If you are not sure if a list item applies to your research, read the appropriate section before selecting a response.

Materials & experimental systems

n/a	Involved in the study
☒	☐ Antibodies
☒	☐ Eukaryotic cell lines
☒	☐ Palaeontology and archaeology
☒	☐ Animals and other organisms
☐	☒ Clinical data
☒	☐ Dual use research of concern
☒	☐ Plants

Methods

n/a	Involved in the study
☒	☐ ChIP-seq
☒	☐ Flow cytometry
☒	☐ MRI-based neuroimaging

Clinical data

Policy information about clinical studies

All manuscripts should comply with the ICMJE guidelines for publication of clinical research and a completed CONSORT checklist must be included with all submissions.

Clinical trial registration: There is no clinical trial element to the study.

Study protocol: N/A

Data collection: N/A

Outcomes: N/A

Plants

Seed stocks: *Report on the source of all seed stocks or other plant material used. If applicable, state the seed stock centre and catalogue number. If plant specimens were collected from the field, describe the collection location, date and sampling procedures.*

Novel plant genotypes: *Describe the methods by which all novel plant genotypes were produced. This includes those generated by transgenic approaches, gene editing, chemical/radiation-based mutagenesis and hybridization. For transgenic lines, describe the transformation method, the number of independent lines analyzed and the generation upon which experiments were performed. For gene-edited lines, describe the editor used, the endogenous sequence targeted for editing, the targeting guide RNA sequence (if applicable) and how the editor was applied.*

Authentication: *Describe any authentication procedures for each seed stock used or novel genotype generated. Describe any experiments used to assess the effect of a mutation and, where applicable, how potential secondary effects (e.g. second site T-DNA insertions, mosiacism, off-target gene editing) were examined.*
